# Vitamin D regulates COVID-19 associated severity by suppressing the NLRP3 inflammasome pathway

**DOI:** 10.1371/journal.pone.0302818

**Published:** 2024-05-15

**Authors:** Bariaa Khalil, Narjes Saheb Sharif-Askari, Shirin Hafezi, Fatemeh Saheb Sharif-Askari, Fatme Al Anouti, Qutayba Hamid, Rabih Halwani

**Affiliations:** 1 Research Institute for Medical and Health Sciences, University of Sharjah, Sharjah, United Arab Emirates; 2 College of Medicine, University of Sharjah, Sharjah, United Arab Emirates; 3 College of Pharmacy, University of Sharjah, Sharjah, United Arab Emirates; 4 College of Natural and Health Sciences, Zayed University, Abu Dhabi, United Arab Emirates; 5 ASPIRE Precision Medicine Research Institute, Abu Dhabi, United Arab Emirates; 6 Department of Clinical Sciences, College of Medicine, University of Sharjah, Sharjah, United Arab Emirates; 7 Meakins-Christie Laboratories, Research Institute of the McGill University Health Center, Montreal, Quebec, Canada; 8 Prince Abdullah Ben Khaled Celiac Disease Research Chair, Department of Pediatrics, Faculty of Medicine, King Saud University, Riyadh, Saudi Arabia; The University of Melbourne at the Peter Doherty Institute for infection and immunity, AUSTRALIA

## Abstract

**Background:**

The role of vitamin D3 (VitD3) in modulating innate and adaptive immunity has been reported in different disease contexts. Since the start of the coronavirus disease-2019 (COVID-19) pandemic, the role of VitD3 has been highlighted in many correlational and observational studies. However, the exact mechanisms of action are not well identified. One of the mechanisms *via* which VitD3 modulates innate immunity is by regulating the NLRP3-inflammasome pathway, being a main underlying cause of SARS-CoV-2-induced hyperinflammation.

**Aims and main methods:**

Blood specimens of severe COVID-19 patients with or without VitD3 treatment were collected during their stay in the intensive care unit and patients were followed up for 29 days. qPCR, western blot, and ELISA were done to investigate the mechanism of action of VitD3 on the NLRP3 inflammasome activation.

**Key findings:**

We here report the ability of VitD3 to downregulate the NLRP3-inflammsome pathway in severe COVID-19 patients. Lower inflammasome pathway activation was observed with significantly lower gene and protein expression of NLRP3, cleaved caspase-1, ASC and IL-1β among severe COVID-19 patients treated with VitD3. The reduction of the inflammasome pathway was associated with a reduction in disease severity markers and enhancement of type I IFN pathway.

**Significance:**

Our data reveals an important anti-inflammatory effect of VitD3 during SARS-CoV-2 infection. Further investigations are warranted to better characterize the ability of VitD3 to control disease pathogenesis and prevent progression to severe states. This will allow for a more efficient use of a low cost and accessible treatment like VitD3.

## Introduction

Severe acute respiratory syndrome coronavirus 2 (SARS-CoV-2) caused the coronavirus disease-2019 (COVID-19) pandemic. Despite the key role of the innate and adaptive immunity in the defense against SARS-CoV-2, the dysregulation in the immune system and the consequent cytokine storm are the main underlying causes behind severe pneumonia and acute respiratory distress syndrome (ARDS) which lead to death in around 40% of infected cases [1]. Importantly, the NLRP3-inflammasome pathway, an important arm of the innate immunity, was reported to contribute to SARS-CoV-2-induced hyperinflammation and was correlated with disease severity [2–7]. The NLRP3 inflammasome is a molecular platform which induces inflammation upon activation, cleavage and release of key inflammatory molecules such as active caspase-1 (CASP-1), IL-1β, and IL-18 [8]. Accordingly, several studies demonstrated targeting inflammasome activation during SARS-CoV-2 to attenuate inflammation by various means such as inflammasome inhibitors, colchicine, microRNAs and long non-coding RNAs in nanomaterials [9–11]. Hence, this pathway stands to be an important therapeutic target worth further investigation in the context of SARS-CoV-2 infection.

The massive improvement in understanding the role of the immune system during the pathogenesis of COVID-19 helped in developing viral treatments, targeted therapies and vaccines which reduced mortality and contained the pandemic. Among the treatment modalities, vitamin D (VitD3) gained impetus among COVID-19 patients as an effective mechanism of population protection. Based on the various immune functions of VitD3, it is expected that deficiency in the hormone is linked to reduced immune response to fight SARS-CoV-2 especially that cross-sectional clinical studies demonstrated that lower serum VitD3 levels are significantly associated with respiratory tract infections [12–15]. In the context of SARS-CoV-2, observational and correlational studies revealed that VitD3 deficiency stood to be an independent risk factor for adverse outcomes of COVID-19 [16, 17]. Accordingly, preliminary data of interventional experiments showed promising results in mitigating COVID-19 severity, shortening hospital stay and enhancing survival rates following VitD3 supplementation [18, 19]. Moreover, correlational analysis of serum biomarkers indicated that VitD3 supplementation might have regulated INOS1, IL-1β, IFNγ, cathelicidin-LL37, and ICAM1 [19]. However, meta-analysis of randomized controlled trials, case control and cohort studies reported inconsistent findings regarding the use of VitD3 supplementation as prophylactic or therapeutic treatment to reduce risk or severity of COVID-19 [20–23]. This is attributed to the large variation in VitD3 supplementation schemes used in different studies and to overlooking confounders that can lead to misleading estimation. Hence, more research on the possible role of VitD3 in influencing the risk and progression of COVID-19 disease is required.

Interestingly, VitD3 was shown to suppress inflammasome activation and IL-1β release in different disease contexts [24–27]. Targeting the inflammasome pathway by VitD3 can either be direct *via* physical interaction between VDR and NLRP3 or indirect whereby VitD3/VDR signaling enhances the antioxidant status of cells and reduces the ROS-mediated activation of the NLRP3 inflammasome [28, 29]. Recently, VitD3 was shown to attenuate hyperinflammation induced by SARS-CoV-2 nucleocapsid protein by inactivating the NLRP3 inflammasome through the VDR-BRCC3 signaling pathway in human bronchial epithelial (HBE) cells and in AAV-Lung-enhanced green fluorescent protein-N-infected mice lungs [30]. Here, we have shown both *in vitro* and using a cohort of COVID-19 hospitalized patients with or without VitD3 treatment, that VitD3 exhibits an anti-inflammatory activity by targeting the NLRP3 inflammasome pathway at both transcriptional and translational levels. Attenuating the NLRP3 inflammasome pathway by VitD3 was accompanied by a decrease in IL-1β, IL-6, IL-17, and D-dimer, reduced hyperinflammation of severely infected COVID-19 patients, as well as correlated with enhanced type I IFN signaling and reduced disease severity.

## Materials and methods

### Publicly available datasets

Publicly available transcriptomic datasets were obtained from the National Center for Biotechnology Information Gene Expression Omnibus (NCBI GEO, http://www.ncbi.nlm.nih.gov/geo) and the European Bioinformatics Institute (EMBL-EBI, https://www.ebi.ac.uk). Analysis of publicly available datasets involved studying whole RNA sequencing data from COVID-19 nasopharyngeal swabs (GSE152075) [31], whole blood (EGAS00001004503) [32], and leukocytes (GEO: GSE157103) [33]. Two studies on COVID-19 samples from BALF and PBMCs were used to obtain single-cell RNA sequencing data regarding the involvement of the NLRP3 inflammasome pathway (GSE145926) [34], and GSE149689 [35]. In addition, three datasets for *in vitro* studies of both humans (GSE208320 and GSE169241) [36, 37] and murine macrophages (GSE182264) [38] infected or transfected with SARS-COV-2 virus spike protein were analyzed. Details of the datasets used are presented in S1 Table. The details of sample isolation, sequencing, and data processing are available at NCBI GEO, along with the protocol of each study.

### COVID-19 patient cohort

Blood specimens were collected as a part of patient routine clinical care from 45 patients with confirmed qPCR for SARS-CoV-2 admitted to intensive care unit at the Rashid Hospital in Dubai between May 2020, and August 2022. Patients who presented with minor respiratory symptoms without need of hospitalization were defined as mildly infected, whereas patients with pneumonia requiring high-flow oxygen therapy or mechanical ventilation were defined as severely infected [39, 40]. VitD3, specifically cholecalciferol, was administered as 50,000 IU weekly for 2–3 weeks to 21 patients based on the national treatment protocols and physician recommendation [39, 40]. The remaining 24 patients did not receive VitD3. The selected patients in the study were adjusted for demographics, comorbidities, and common laboratory inflammatory markers of COVID-19 severity, and the use of any kind of interferon-based therapy such as IFNβ1a or IFNβ1b. Table 1 illustrates the laboratory and clinical test results upon admission. The serum 25-hydroxyVitD3 [25(OH)D] level was not reported given it was not required for COVID-19 national clinical management protocols [39, 40]. This study was done under the ethical approval obtained from the Dubai Scientific Research Ethics Committee (DSREC), Dubai Health Authority at Rashid Hospital (DSREC-12/2020_02). All participants provided written informed consent. All methods were performed in accordance with the relevant guidelines (Declaration of Helsinki and the Belmont Report) and regulations (DSREC rules) following CDC recommended precautions for safe collection, handling and testing of biological fluids [41].

**Table 1:** Baseline clinical characteristics of the study population.

Variables	VitD3 treated patients (n = 21)	Untreated patients (n = 24)	p-value
Age, yrs	63 (53–80)	59 (43–68)	0.144
Male sex	19 (90)	20 (83)	0.482
BMI	29.5 (24.7–32.5)	27.3 (24–33.3)	0.904
Diabetes mellitus	8 (38)	12 (50)	0.264
Hypertension	8 (38)	5 (21)	0.280
**Baseline laboratory data (normal range)**			
D-Dimer (0–0.5 μg/mL)	1.6 (0.9–2.2)	4.3 (1.9–7.4)	**0.005**
Cytokines levels*			
Plasma IL-6 (pg/ml)	15.3 (11.1–22.3)	430 (125–765)	**0.01**
Plasma IL-17 (pg/ml)	22 (18.3–30.6)	332 (96.7–606.8)	**0.017**
**COVID-19 Supportive Medication**			
Tocilizumab	3 (14)	4 (17)	0.826
IFN-α/β	1 (4.8)	2(8.3)	0.632
Outcome*			
Length of ICU stay, median, days	20 (12.5–28)	27 (21.3–28)	**0.018**

Data are n (%) or median (IQR). Abbreviation: BMI, body mass index, ICU, Intensive care unit. *P-values for plasma cytokine levels and length of stay were adjusted for patients age, male sex, and body mass index. All the patients were administered systemic corticosteroids, such as dexamethasone or methylprednisolone.

### In vitro treatment

Ficoll-Paque was used to isolate human peripheral blood mononuclear cells (PBMCs) from the peripheral blood of 3 healthy control donors. PBMCs from each donor were resuspended in complete RPMI-1640 media and plated into non-adherent 12-well plates at a density of 1 ×10^6^ cells per well overnight. On the day of the treatment, cells were primed with 150ng/ml lipopolysaccharide (LPS) (O111:B4) from Escherichia coli (Sigma-Aldrich, Merck, Darmstadt, Germany) for 3 hours. Then, 10nM SARS-CoV-2 spike protein (S1+S2 ECD, His Tag) (Sino Biological, 40589-V08B1) was added for 16 h after which the cells were harvested for lysate preparation, and the supernatants were collected for cytokine/chemokine release by ELISA. For VitD3 treatment conditions, 50nM calcitriol (Sigma-Aldrich, USA) was added during the priming step along with LPS prior to inflammasome activation by SARS-CoV-2 spike protein. MCC950 (Tocris Bioscience, CAS-256373-96-3), a selective and highly potent NLRP3 inhibitor was used at 10uM as a positive control.

### Protein expression by western blot

Pellets from whole blood of COVID-19 patients or PBMCs of healthy donors were obtained by centrifugation at 14000*g* for 20 min at 4°C. The protein concentrations were measured using the BCA protein assay reagent kit (Thermo-Scientific Pierce BCA Protein Assay Kit). Cells were lysed using 1X RIPA Buffer supplemented with 1 × Protease Inhibitor Cocktail (Sigma-Aldrich, USA) and 1 mM phenylmethylsulfonyl fluoride (Sigma-Aldrich, USA). 20–40 micrograms total proteins were separated using 8%, 10% or 12.5% gels. The proteins were transferred onto a nitrocellulose membrane (Bio-Rad), blocked in skimmed milk for 1 h at room temperature, incubated overnight at 4°C with antibodies specific to NLRP3 (Adipogen, AG-20B-0014-C100), cleaved CASP-1 (Cell Signaling, 4199), ASC (Santa Sruz, sc-514414) and GAPDH (Cell Signaling, 2118). The blots were developed using the Clarity Western ECL Substrate (Bio-Rad) in the ChemiDoc Touch Gel Imaging System (Bio-Rad). Image J software was used to detect and quantify the protein bands.

### IL-1β enzyme-linked immunosorbent assay

IL-1β cytokine concentrations were determined in whole blood samples using commercially available human ELISA kit (Human IL-1 beta/IL-1F2 DuoSet ELISA (DY201-5), R&D Systems). Assays were performed following the manufacturer’s instructions. All samples were measured in duplicates.

### Gene expression assay using qRT-PCR

Total RNA from whole blood of COVID-19 patients was isolated using Trizol reagent according to the manufacturer’s instructions (Invitrogen, Carlsbad, CA) [42]. Complementary DNA (cDNA) converted from 200ng of RNA using the High-Capacity cDNA Reverse Transcription Kit (Applied Biosystems) according to the manufacturer’s protocol. For cDNA amplification, 5 × Hot FirePol EvaGreen qRT-PCR SuperMix (Solis Biodyne, USA) was used, and qRT-PCR was performed in QuantStudio 3 Real-Time PCR System (Applied Biosystems) [43]. Primer sequences for NLRP3, CASP-1, PYCARD, IL-1β and IL-18 used in qRT-PCR are deposited in table in S2 Table.

### Analysis procedures

Bioinformatic analyses were conducted in the following manner. For the single-cell investigations, we utilized the published processed data. The authors used the Model-based Analysis of Single-cell Transcriptomics (MAST) algorithm in Seurat v3 to identify differentially expressed genes (DEGs) and determine the log2 fold change. For RNA-seq datasets, we processed the data using the Bioconductor package limma-voom and presented the results as log2 counts per million (log CPM). In our study, we used log-transformed normalized intensities in Linear Models for MicroArray data (LIMMA) analyses to identify differentially expressed genes between the diseased and control groups. The default Benjamini-Hochberg correction was employed for multiple testing. Furthermore, a Gene Set Enrichment Analysis (GSEA) was performed on the nasopharyngeal swab dataset (GSE152075) to show the enrichment of the inflammasome pathway (REACTOME_THE_NLRP3_INFLAMMASOME) obtained from the GSEA database (https://www.gsea-msigdb.org) [44]. For the pathway analysis, expressed genes in the Reactome of NLRP3 Inflammasome pathway were examined using Gene Set Enrichment Analysis (GSEA). This method first ranks the expression value of each gene by signal-to-noise ratio (|S2N|), which estimates the difference in gene expression between two experimental settings, in this case, COVID-19 and the negative control group. Subsequently, it calculates the Normalized Enrichment Score (NES) by evaluating the ranked list of genes within a specified set (e.g., Reactome NLRP3 Inflammasome) using running-sum statistics. Additionally, it identifies the top or bottom leading-edge subsets, which serve as the core of the given set and account for the enrichment signals [44].

Correlation analysis was performed using either Pearson or Spearman correlation, depending on the data’s skewness. Correlation was adjusted with the Bonferroni method. The statistical analyses were conducted using R software (version 3.0.2), SPSS 28.00 (SPSS Inc., Chicago, IL, USA), and Prism (version 8; GraphPad Software). Statistical results obtained through two-sided tests with a p-value less than 0.05 were considered significant.

### Ethics statement

No experiments on animals were performed for this manuscript. For human subjects, written and informed consents were obtained from all study participants prior to inclusion. This study was approved by Dubai Scientific Research Ethics Committee (DSREC).

## Results

### NLRP3 inflammasome pathway is upregulated during SARS-CoV-2 infection and is associated with disease severity

**Fig 1:**
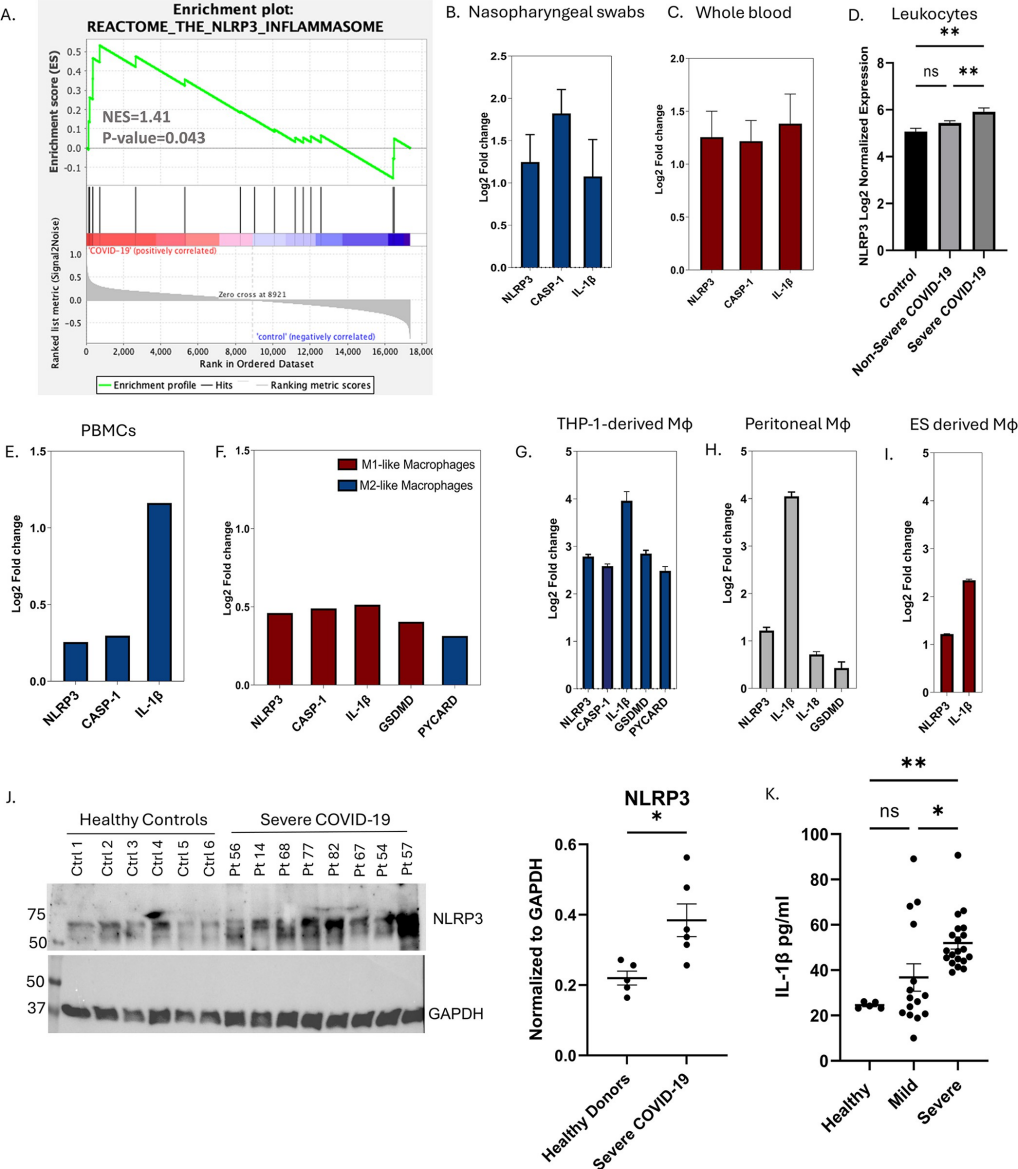
NLRP3 inflammasome pathway is upregulated during SARS-CoV-2 infection and is associated with disease severity. (A) Gene Set Enrichment Analysis (GSEA) on nasopharyngeal swab dataset (GSE152075) showing the enrichment of the NLRP3 inflammasome pathway (REACTOME_THE_NLRP3_INFLAMMASOME). The genes shown in the transcriptomic analysis in Fig 1B–1I are the significantly upregulated genes out of 6 screened genes involved in the NLRP3 inflammasome pathway. (B) RNAseq profiles of the nasopharyngeal swabs (GSE152075) of SARS-CoV-2 infected individuals (430) and healthy controls (54) showed a log2 fold change in NLRP3 (1.24), CASP-1 (1.82) and IL-1β (1.07). (C) Increased expression of important players in the NLRP3 inflammasome pathway (NLRP3 (1.25), CASP-1 (1.21) and IL-1β (1.38)) in whole blood of severe transcriptome of COVID-19 patients (20) as compared to control subjects (10) using RNAseq data EGAS00001004503. (D) RNAseq performed on leukocyte dataset (GEO: GSE157103) with 37 severe and 51 non-severe COVID-19 patients with severity based on ICU admission. (E) Single-cell RNA sequencing dataset (GSE149689) showing log2 fold change in the expression of NLRP3 (0.25), CASP-1 (0.3) and IL-1β (1.2) in monocytes of PBMCs from severe and mildly infected COVID-19 patients. (F) Log2 fold changed of NLRP3 (0.4), CASP-1 (0.5) and IL-1β (0.55) using single-cell RNA sequence in M1-like and M2-like macrophages in the BALF obtained from 6 severe and 3 moderate COVID-19 patients (GSE145926), as well as 3 healthy controls. (G) GSE182264 data of murine peritoneal macrophages transfected with the Spike protein (n = 5) in comparison to control samples (n = 5). (H) GSE208320 compared data between two samples of THP-1-derived macrophages transfected with the SARS-CoV-2 spike protein and two control samples. (I) GSE169241 datasets examined SARS-CoV-2 infected human embryonic stem cell (ES) derived macrophages in three SARS-CoV-2 infected and three control samples. Results are presented as log2 fold change ± SE of gene expression between cases and controls. (J) The protein levels of NLRP3 in whole blood of severe COVID-19 patients as compared to healthy controls. (K) The plasma levels of IL-1β in severe COVID-19 patients as compared to healthy controls and mild cases. Results are presented as mean (± SEM) and relative to the control group. Statistic test: comparison was done using unpaired t-test or one-way ANOVA *P<0.05, **P<0.01, ***P<0.001).

The upregulation of NLRP3 inflammasome pathway during SARS-CoV-2 infection and its link with disease severity were evaluated using publicly available transcriptomic datasets (Table in S1 Table and data in S1 File). To evaluate the involvement of the NLRP3 pathway, GSEA was carried out on the COVID-19 nasal swab dataset (GSE152075). Compared to uninfected controls, infection with SARS-CoV-2 resulted in an enriched NLRP3 pathway (Normalized Enrichment Score, NES 1.41, P-value = 0.043) (Fig 1A). First, we looked at the expression of NLRP3 inflammasome pathway related genes in nasopharyngeal swabs (GSE152075), whole blood (EGAS00001004503), and leukocytes (GSE157103) of COVID-19 patients. The nasopharyngeal swabs of 430 SARS-CoV-2 infected individuals showed log2 fold change of NLRP3 (1.24), CASP-1 (1.82) and IL-1β (1.07) when compared to 54 healthy controls (Fig 1B). Despite the severity status of the patients, these results confirmed the involvement of the NLRP3 inflammasome pathway in the pathogenesis of SARS-CoV-2 infection. The RNAseq data on whole blood (EGAS00001004503) of 10 controls, 20 severe and 19 mild COVID-19 patients showed log2 fold change in NLRP3 (1.25), CASP-1 (1.21) and IL-1β (1.38) in severely infected patients only. This reflected the association between the severity status of SARS-CoV-2 infection and the upregulation of NLRP3 inflammasome pathway (Fig 1C). Further validation on leukocytes of COVID-19 patients (GEO: GSE157103) classified as severe (37) or non-severe (51) based on ICU admission, demonstrated that SARS-CoV-2 infected patients admitted to the ICU exhibited a significant increase in the expression of NLRP3 as compared to non-severe COVID-19 patients (Fig 1D).

However, analysis of single-cell RNA sequencing datasets obtained from bronchioalveolar fluid (BALF) and PBMC samples of COVID-19 patients demonstrated that the activation of the inflammasome pathway occurs in monocytes and macrophages but not in other immune cells including B-cells, CD4^+^ and CD8^+^ T-cells or natural killer cells [34, 35]. In the first study, Lee JS *et al*. (GSE149689) performed single sequencing on peripheral blood mononuclear cells (PBMCs) isolated from 5 flu, 8 COVID-19 patients, and 4 healthy controls [35]. The log2 fold change of differentially expressed genes (DEGs) in monocytes within PBMCs of severe and mild COVID-19 patients confirmed the log2 fold change observed in NLRP3 (0.22), CASP-1 (0.29) and IL-1β (1.16) during severe stages of SARS-CoV-2 infection (Fig 1E). In the second study conducted by Liao *et al*. (GSE145926), BALF was obtained from 6 severe and 3 moderate COVID-19 patients, as well as 3 healthy controls [34]. The macrophages were clustered into four groups based on the expression of differentiation markers. Group one and two were classified as M1-like macrophages, while group three was classified as M2-like macrophages. Log2 fold changes generated for each group of macrophages relative to the total macrophage population showed an increase in NLRP3 (0.46), IL-1β (0.51), CASP-1 (0.48) and GSDMD (0.4) in the M1-like macrophages as compared to M2-like macrophages and this increase is higher in severely infected patients compared to non-severe groups (Fig 1F). This reflects on the association between the inflammatory nature of the M1 group and the inflammasome pathway and emphasizes its role in inducing inflammation during SARS-CoV-2 infection.

In addition, three datasets for *in vitro* studies of macrophages were analyzed. The first dataset (GSE208320) compared two samples of THP-1-derived macrophages transfected with the SARS-CoV-2 spike protein and two control samples (Fig 1G). Log2 fold change was detected in NLRP3 (2.78), ASC/PYCARD (2.48), CASP-1 (2.58), IL-1β (3.95) and GSDMD (2.84). The second dataset (GSE182264) analyzed murine peritoneal macrophages transfected with the spike protein (n = 5) in comparison to control samples (n = 5) and showed log2 fold change in NLRP3 (1.21) and IL-1β (4.04) (Fig 1H). The second dataset (GSE169241) examined SARS-CoV-2 infected human embryonic stem cell (ES) derived macrophages in three SARS-CoV-2 infected and three control samples (Fig 1I). Log2 fold changes in NLRP3 (1.21) and IL-1β (2.33) were detected. Collectively, the *in vitro* data highlighted the ability of the SARS-CoV-2 spike protein to activate the inflammasome pathway as a mechanism *via* which it leads to hyperinflammation. Hence, the NLRP3 inflammasome pathway is a confirmed underlying mechanism implicated in the pathogenesis of SARS-CoV-2 infection. Accordingly, the NLRP3 pathway stands to be an important target to reduce hyperinflammation especially in severely infected patients who were shown to exhibit higher levels of NLRP3 inflammasome activation compared to mild cases. Using our cohort of COVID-19 patients, we validated the findings of publicly available dataset and confirmed the involvement of the inflammasome pathway in COVID-19 pathogenesis especially in severely infected patients. Significantly higher protein levels of NLRP3 were detected in severe SARS-CoV-2 patients as compared to healthy donors (Fig 1J and S1 Fig in S2 File). This was further associated with a significant release of proinflammatory IL-1β in the plasma of severe cases when compared to healthy donors and mild cases (Fig 1K). Hence, our cohort confirms the link between the upregulation and activation of NLRP3 inflammasome pathway and patients’ severity status.

### VitD3 attenuates NLRP3 inflammasome pathway in the blood of severe COVID-19 patients

**Fig 2:**
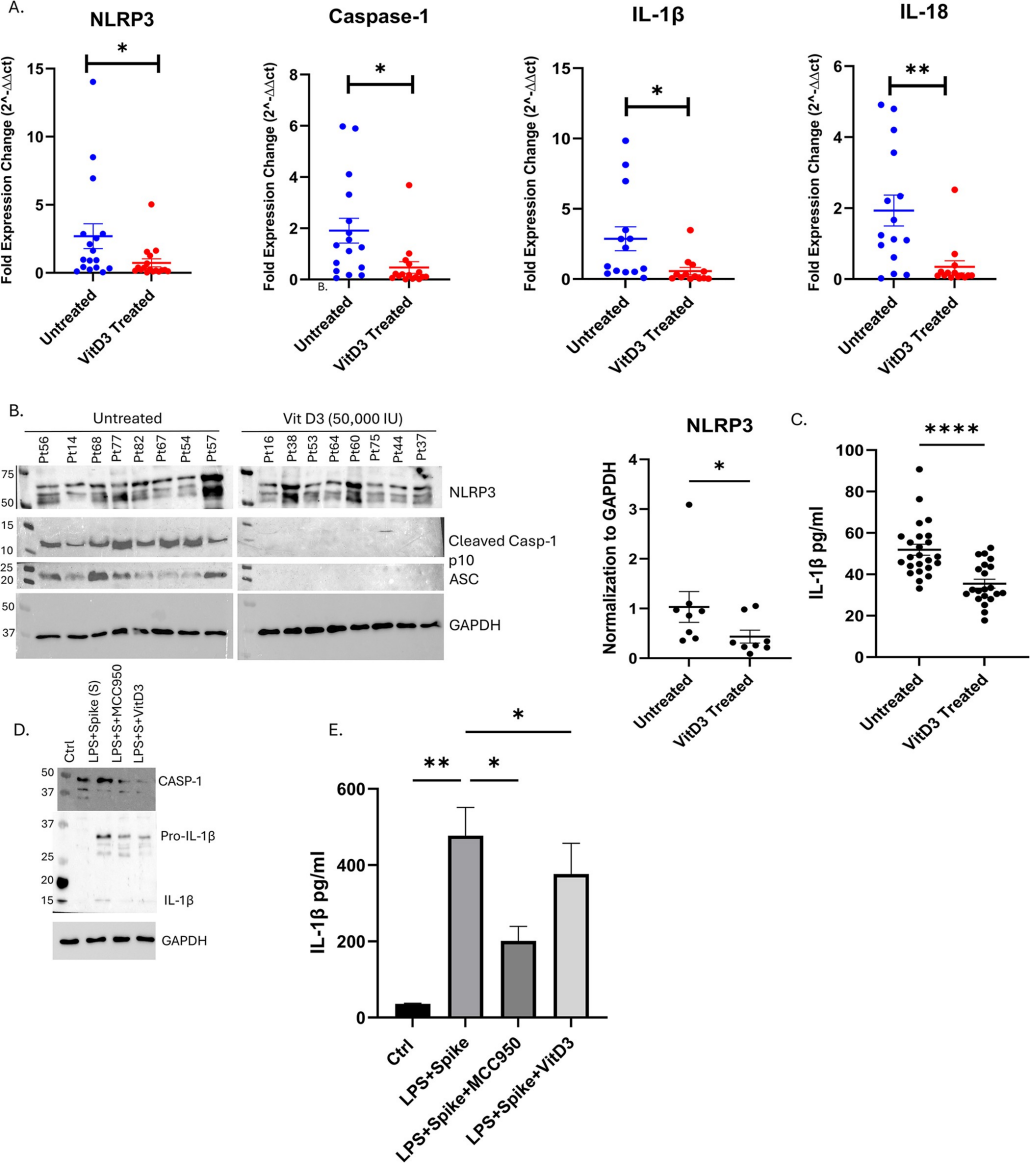
VitD3 attenuates NLRP3 inflammasome pathway in the blood of severe COVID-19 patients and in PBMCs treated with SARS-CoV-2 spike protein. (A) The mRNA levels of NLRP3, ASC, CASP-1, IL-1β and IL-18 in whole blood of VitD3 treated and untreated COVID-19 patients.18S was used as a house keeping gene for qRT-PCR. (B) The protein levels of NLRP3, ASC and cleaved CASP-1 in whole blood of VitD3 treated and untreated COVID-19 patients. (C) The plasma levels of IL-1β in VitD3 treated (n = 21; 19 males and 2 females) and untreated severe COVID-19 patients (n = 24; 20 males and 4 females). Results are presented as mean (± SEM) and relative to the untreated group. The protein level of CASP-1 and IL-1β in VitD3 (50nM of calcitriol) treated PBMCs. (D) Protein levels of CASP-1 and IL-1β in VitD3 and/or LPS primed, and SARS-CoV-2 spike (50nM of calcitriol and/or 100ng/ml LPS and10nM Spike for 16 h) treated PBMCs. (E) The level of IL-1β in culture supernatant in VitD3 and/or spike treated PBMCs. Results are presented as mean (± SEM) and relative to the control PBMCs. Statistic test: comparison was done using unpaired t-test or one-way ANOVA *P<0.05, **P<0.01, ***P<0.001).

VitD3 was reported in different cross-sectional, randomized controlled trials, case control and cohort studies to be a promising low cost and accessible supplement in mitigating COVID-19 severity, shortening hospital stay and enhancing survival rates [19, 22, 23]. Interestingly, a recent study showed using *in vitro* and *in vivo* mouse model that VitD3 was able to attenuate the hyperinflammation induced by SARS-CoV-2 nucleocapsid protein by inactivating the NLRP3 inflammasome through the VDR-BRCC3 signaling pathway. Hence, we investigated whether VitD3 treatment can reduce the NLRP3 inflammasome pathway in a cohort of severe COVID-19 patients. For this, we have measured the expression of the different players implicated in the NLRP3 inflammasome pathway including NLRP3, CASP-1, ASC, IL-1β and IL-18 in whole blood of severe COVID-19 patients treated or not with 50,000 IU cholecalciferol once per week for 2–3 weeks during their stay at ICU. A total of 45 patients with severe SARS-CoV-2 infection were divided into two groups: VitD3 treated group (n = 21 patients) and untreated group (n = 24 patients). The two groups had similar demographics, comorbidities, and clinical laboratory tests (Table 1). Previously our group reported that the expression of the VDR at both the mRNA and protein levels was higher in blood of the VitD3 treated group [45]. Importantly, VitD3 was capable of significantly reducing mRNA levels of NLRP3 (P<0.05), CASP-1 (P<0.05), IL-1β (P<0.05) and IL-18 (P<0.01) in the whole blood of treated compared to untreated patients (Fig 2A). This was further accompanied by significant reduction of NLRP3 (P<0.05), cleaved CASP-1 and ASC proteins as well as plasma IL-1β release (P<0.001) (Fig 2B, 2C and S2 & S3 Figs in S2 File). This suggests that VitD3 treatment ameliorated the activation of the NLRP3 inflammasome pathway in severely infected patients at the transcription and the translational level.

### VitD3 attenuates SARS-CoV-2 spike protein induced NLRP3 inflammasome pathway activation

Furthermore, we examined the regulatory effect of VitD3 on the activation of NLRP3 inflammasome pathway by treating PBMCs isolated from healthy subjects with 50 nM VitD3 (calcitriol) and/or LPS and wild type SARS-CoV-2 spike protein. MCC950, a selective and highly potent NLRP3 inhibitor was used as a positive control. LPS priming and activation with SARS-CoV-2 spike protein were shown to upregulate CASP-1, whereas VitD3 and MCC950 reversed this effect at the protein level (Fig 2D and S4 Fig in S2 File). This was further accompanied by a reduction in the upregulation and cleavage of IL-1β, the main inflammatory product of the NLRP3 inflammasome pathway. This reflects the inhibition of CASP-1 activation upon VitD3 and MCC950 treatment. Consequently, the release of IL-1β in cell culture supernatant was shown to significantly decrease in the presence of VitD3 or MCC950 (Fig 2E).

### Downregulating the NLRP3 inflammasome pathway is associated with an enhanced Type 1 IFNs signaling

**Fig 3:**
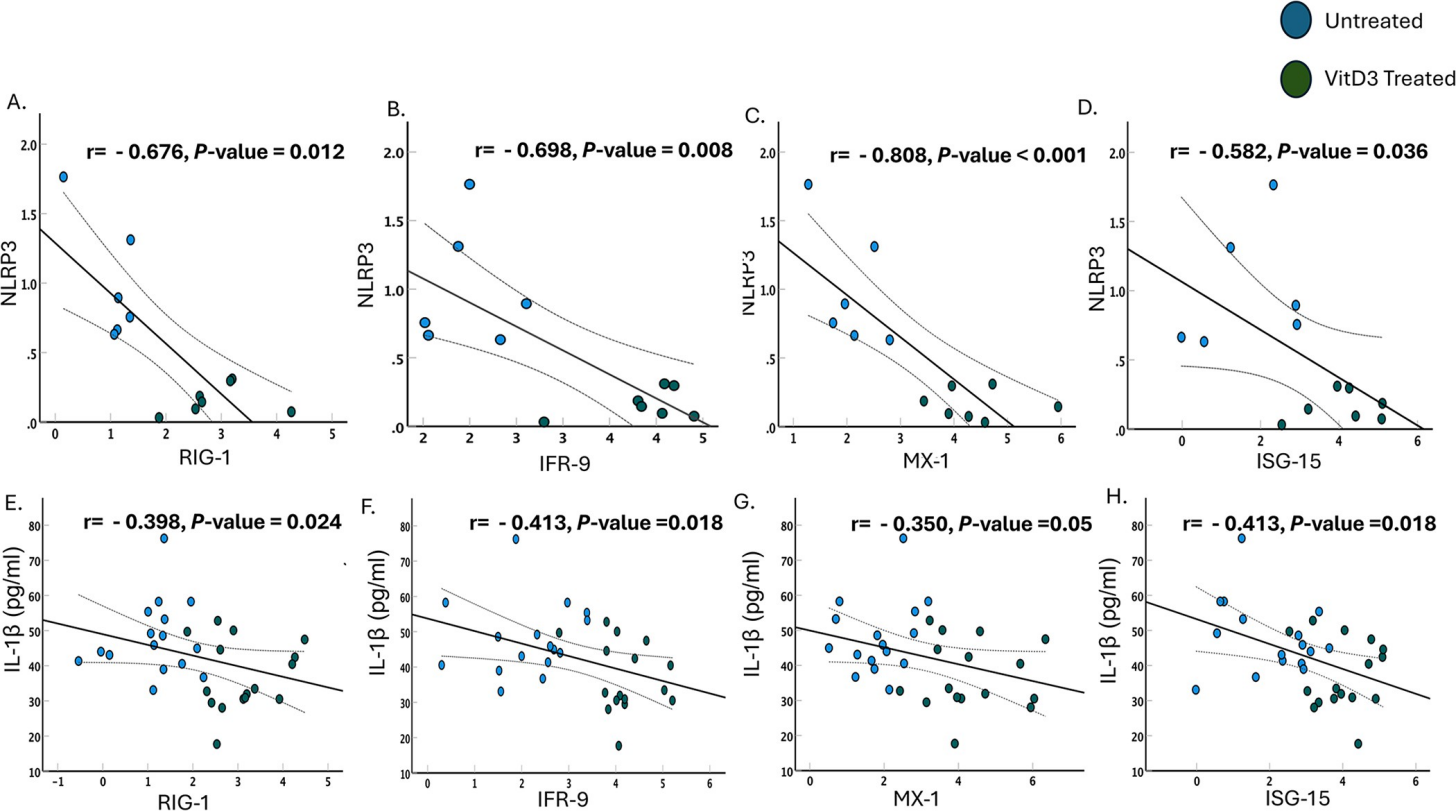
Downregulating the NLRP3 inflammasome pathway is associated with an enhanced Type 1 IFNs signaling. Lower levels of NLRP3 proteins in VitD3 treated COVID-19 patients were associated with enhanced mRNA expression of RIG-1/MDA-5 signaling pathway, interferon regulatory protein IRF-9 and interferon stimulating genes MX-1 and ISG-15 as compared with untreated COVID-19 patients (A-D). Lower levels of plasma IL-1β in VitD3 COVID-19 treated patients were associated with enhanced RIG-1/MDA-5 signaling pathway, interferon regulatory protein IRF-9 and interferon stimulating genes MX-1 and ISG-15 as compared with untreated COVID-19 patients (E-H). Statistical tests: Pearson or Spearman correlation with P value of <0.05 considered significant.

Optimal IFN and inflammasome activation are two important events of a healthy immune reaction required to limit SARS-CoV-2 infection. However, aberrant activation of these two pathways leads to hyperinflammation and contributes to COVID-19 severity. SARS-CoV-2 is known to employ several viral proteins that block IFN signaling pathways and aid the virus to evade host’s innate immunity. Our group showed previously the ability of VitD3 to enhance type I IFN signaling in severely infected COVID-19 patients [45]. Hence, we looked for a possible association between the NLRP3 inflammasome pathway activation and IFN signaling in our cohort of COVID-19 patients admitted to ICU in the presence and absence of VitD3 administration. In VitD3 treated COVID-19 patients, the reduction in the protein levels of NLRP3 and IL-1β were associated with a significant increase in the mRNA levels of RIG-1 (P = 0.006 and P = 0.012 for Fig 3A and 3E, respectively), IRF-9 (P = 0.004 and P = 0.009 for Fig 3B and 3F, respectively), Mx-1 (P<0.001 and P = 0.025 for Fig 3C and 3G, respectively) and interferon stimulating genes-15 (ISG-15) (P = 0.018 and P = 0.009 for Fig 3D and 3H, respectively). Our results revealed that VitD3 reduces hyperinflammation during severe SARS-CoV-2 infection not only by downregulating the NLRP3 inflammasome pathway, but also by enhancing the anti-viral type I IFN signaling. Regulating these two major events of innate immunity by VitD3 attenuated disease severity markers among severe COVID-19 patients.

### VitD3 regulation of the NLRP3 inflammasome pathway is associated with a reduction in disease severity markers

**Fig 4:**
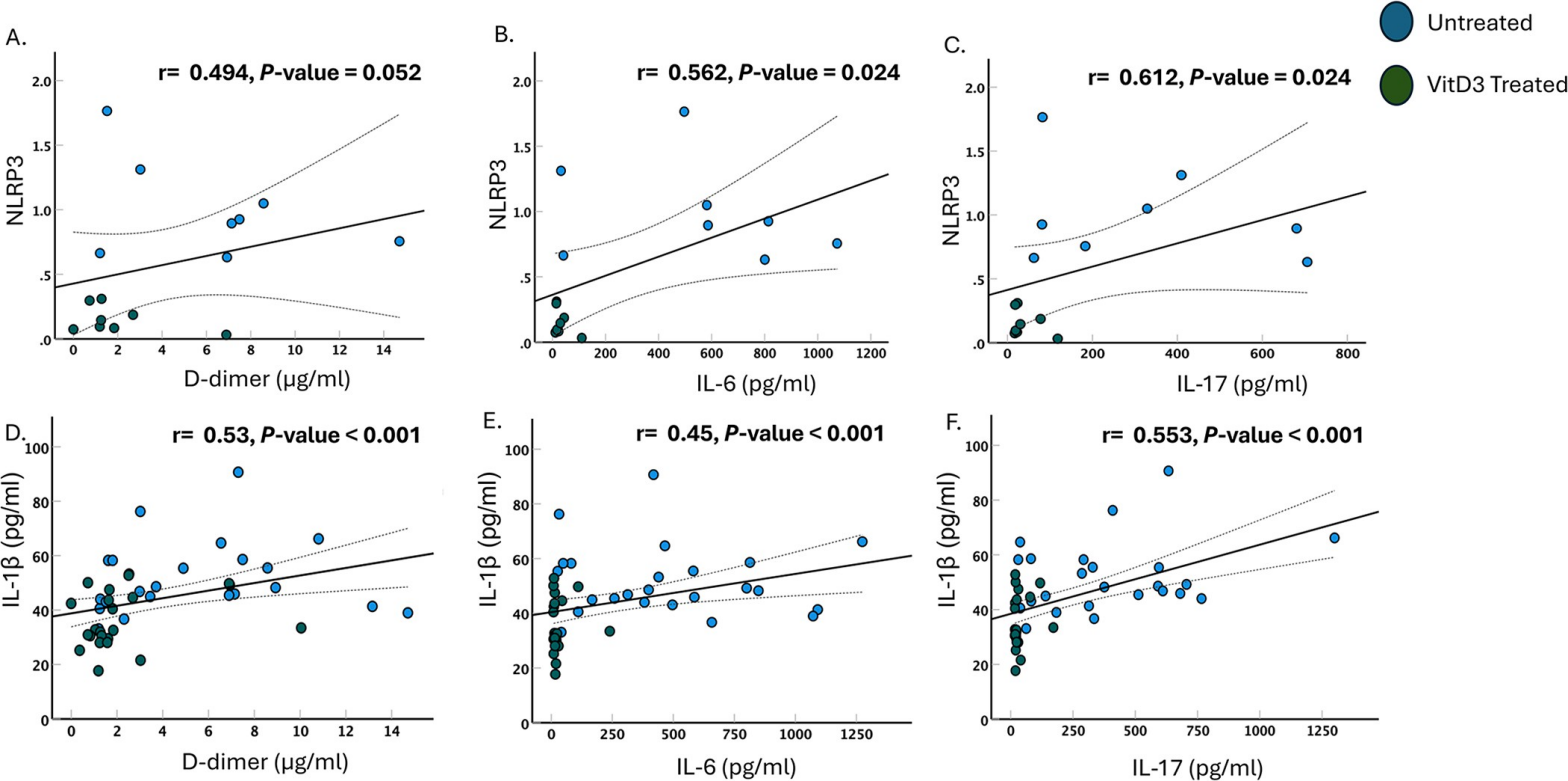
VitD3 regulation of the NLRP3 inflammasome pathway is associated with a reduction in disease severity markers. Lower levels of NLRP3 proteins in VitD3 treated COVID-19 patients were associated with lower levels of hyperinflammatory markers such D-dimer, IL-6 and IL-17 detected by ELISA as compared with untreated COVID-19 patients (A-C). Lower levels of plasma IL-1β in VitD3 COVID-19 treated patients were associated with lower levels of hyperinflammatory markers such D-dimer, IL-6 and IL-17 as compared with untreated COVID-19 patients (D-F). Statistical tests: Pearson or Spearman correlation with P value of <0.05 considered significant.

SARS-CoV-2 virus activation of the inflammasome pathway was reported to be linked to disease severity in long-COVID patients [2, 3, 30]. Lung tissues, peripheral blood mononuclear cells and monocytes of severe COVID-19 patients displayed increased NLRP3 activation and increased levels of IL-1β [2, 46, 47]. The association between the NLRP3 inflammasome pathway and disease severity markers were assessed in the presence and absence of VitD3 among severe COVID-19 patients. In VitD3 treated COVID-19 patients, the reduction in the levels of NLRP3 and IL-1β were associated with a reduction in D-dimer (P = 0.052 and P<0.001 for Fig 4A and 4D, respectively), IL-6 (P = 0.024 and P<0.001 for Fig 4B and 4E, respectively), and IL-17 (P = 0.024 and P<0.001 for Fig 4C and 4F, respectively). Our results confirm the contribution of the NLRP3 inflammasome pathway to COVID-19 severity and the ability of VitD3 to attenuate hyperinflammation *via* downregulating the NLRP3 inflammasome pathway.

## Discussion

**Fig 5:**
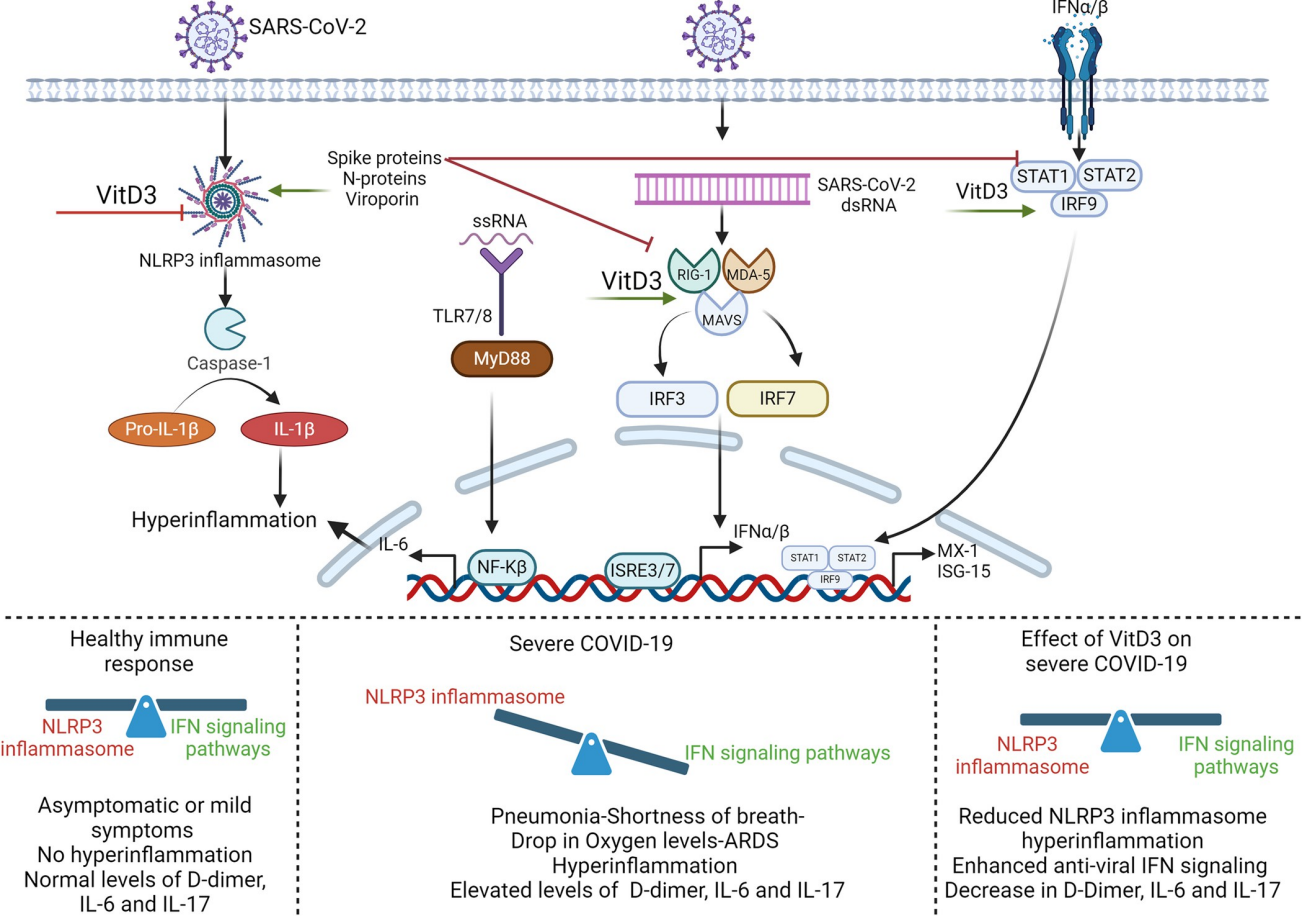
Graphical illustration of main findings of the study. The NLRP3 inflammasome pathway and associated hyperinflammation markers as determined by IL-1β, IL-6, IL-17 and D-Dimer are attenuated upon VitD3 treatment in severe COVID-19 patients. This is associated with an enhancement of the anti-viral immune response as detected by the ability of VitD3 to enhance RIG-1/MDA-5 and JAK-STAT signaling pathways along with the production of ISGs like MX-1 and ISG-15.

In our study, we have demonstrated that one of the possible anti-viral mechanisms of VitD3 against SARS-CoV-2 infection could be attributed to the reduction in the activation of host NLRP3 inflammasome pathway. This finding was demonstrated both *in vitro* and *in vivo* using a cohort of COVID-19 hospitalized patients. Targeting the NLRP3 inflammasome pathway by VitD3 reduced the induced hyperinflammation and attenuated the severity of SARS-CoV-2 infection by reducing IL-1β, IL-17, IL-6, and D-dimer (Fig 5).

Inflammasome activation is one of the most detrimental inflammatory pathways in pulmonary inflammatory conditions [48, 49]. Inflammasomes are cytosolic oligomeric protein complexes composed of NLRs or absent in melanoma 2-like receptors (AIM2), adapter protein apoptosis-associated speck-like protein containing a CARD (ASC), and pro-CASP-1 [50, 51]. Various proteins such as NLRP1, NLRP3 and NLRC4, AIM2, pyrin, NLRP6, NLRP7, NLRP12, retinoic acid-inducible gene I (RIG-I; also known as DDX58) and interferon-γ (IFNγ)-inducible protein 16 (IFI16) are capable of assembling inflammasomes and activating CASP-1 [8]. However, the NLRP3 inflammasome (also called NALP3, PYPAF1, or cryopyrin) is the best characterized, and was reported to contribute to the pathogenesis of several inflammatory diseases [50, 52, 53]. Inflammasomes are assembled and activated upon the binding of pathogen-associated molecular patterns (PAMPs) and damage-associated molecular patterns (DAMPs) to their respective type of pathogen recognition receptors (PRRs) [54]. The activation of CASP-1 by inflammasomes enables it to cleave pro-IL-1β and pro-IL-18 into their active forms (IL-1β and IL-18) [52, 55].

SARS-CoV-2 virus induces the activation of the NLRP3 inflammasome pathway by various viral structural components either directly or indirectly (15). For instance, the spike S1 protein can activate the NLRP3 inflammasome *via* JAKs/STAT3 or Akt-MAPK-AP1 axis signaling pathway and leads to IL-1β and IL-18 secretion [56–60]. Another reported mechanism that activates NLRP3 inflammasome is induced by SARS-CoV-2 viroporin proteins which act as membrane ion channels and facilitate Ca^2+^ flux, ROS production and mitochondrial DNA oxidation [21, 61]. Mitochondrial DNA released through mitochondrial-permeability-pore binds and activates NLRP3 inflammasome [62, 63]. Direct viral protein (N-protein (2-N) and 1-3a) interaction with components or regulatory proteins of the NLRP3 pathway has also been shown to agonize the inflammasome activation with their effect reversed by the specific NLRP3 inhibitor, MCC950, and by the caspase-1 inhibitor Ac-YVAD-cmk [64–66]. This was further associated with IL-1β and IL-6 release which accelerated lung injury and death in mouse models. Aside from the direct interaction with the inflammasome, 2-N, 2-S, 2-7a, 1-3a & 2-3a proteins can also increase NF-κB-dependent transcription of pro-inflammatory cytokines such as IL-18 and IL-1β [67].

Analysis of publicly available datasets (Fig 1A–1I) showed that the NLRP3 inflammasome pathway is an important biomarker for severity. This was revealed using whole RNA sequencing on whole blood samples and nasopharyngeal swabs as well as using single cell RNA sequencing on BALF and PBMCs of SARS-CoV-2 infected patients. Furthermore, transcriptomic analysis of *in vitro* mouse and THP-1 derived macrophages treated with SARS-CoV-2 spike protein, showed log2 fold change elevation in NLRP3 and IL-1β at the mRNA level. Additionally, using a cohort of COVD-19 patients with different disease severity status, the main inflammatory cytokine of the NLRP3 inflammasome pathway, IL-1β, was shown to be significantly upregulated in severe COVID-19 patients when compared to asymptomatic patients. Thus, targeting inflammasome activation is imperative to ameliorate SARS-CoV-2 induced hyperinflammation.

Numerous studies have demonstrated that VitD3 can inhibit the NLRP3 inflammasome and reduce associated inflammation. Our *in vitro* experiments on PBMCs from healthy donors treated with wild type SARS-CoV-2 spike protein following priming with LPS, showed downregulation of the NLRP3 inflammasome, CASP-1 and IL-1β upon administration of VitD3. Interestingly, this finding was as well observed in the whole blood of VitD3 treated severe COVID-19 patients admitted to ICU. VitD3 was able to reduce NLRP3 inflammasome pathway related genes at both transcriptional and translational levels, and significantly reduced the release of inflammatory IL-1β. Our findings go in parallel with a recent study which showed using HBE‐N cells and a murine model of AAV-Lung-enhanced green fluorescent protein-N-infected lungs, that VitD33 can attenuate the hyperinflammation induced by SARS-CoV-2 *via* inactivating the NLRP3 inflammasome pathway [30]. The ability of VitD3 to target the NLRP3 inflammasome pathway especially in severe cases is important in mitigating hyperinflammation and reducing severity-related markers such IL-6, IL-17, and D-dimer.

Type I IFN signaling and NLRP3 inflammasome pathway are two essential events of innate immunity optimally needed to limit SARS-CoV-2 infection. Suppression of type I IFN signaling pathway and exacerbation of NLRP3 inflammasome activation enhance the hyperinflammation and severity of COVID-19 [2, 68–71]. Our group previously showed that VitD3 enhances type I IFN signaling in the same cohort of COVID-19 patients. This was specifically accompanied by higher activity levels of RIG-1/MDA-5 and JAK-STAT signaling pathways as well as significantly higher gene and protein levels of antiviral interferon stimulating genes (ISGs) such as MX-1 and ISG-15 in the whole blood and saliva of VitD3 treated COVID-19 patients [45]. Our correlation analysis revealed that upon VitD3 treatment of severely infected SARS-CoV-2 patients, the downregulation of the NLRP3 inflammasome pathway as detected by a reduction in NLRP3 and IL-1β protein levels, was associated with an enhancement of type I IFN signaling as detected at the gene level of the RIG-1/MDA-5 signaling pathways and ISGs. Enhancing the anti-viral immune response while at the same time decreasing hyperinflammation was capable of ameliorating disease severity by reducing IL-6, IL-17, and D-dimer. It is worth highlighting that the cross talk between innate immune signaling pathways varies in different infectious diseases. For instance, during Toxoplasma gondii (T.gondii) infection, the role of IFN-1 is rather harmful and the activation of the inflammasome signaling by PAMPs of T.gondii generated protective immunity against bacterial invasion by suppressing type I IFN production [72]. Hence, in the context of SARS-CoV-2 infection, the fine tuning and tight regulation of the NLRP3 inflammasome pathway and type I IFN signaling is key to avoid hyperinflammation and disease progression to severe states.

While our cohort showed a potential effect of VitD3 on the NLRP3 inflammasome pathway associated with a reduction in disease severity markers and enhancement of type I IFN signaling pathway, other double-blind controlled trials and open-labeled studies showed no significant effect of VitD3 supplementation on median length of hospital stay, the need for mechanical ventilator and mortality [73–76]. Aside from confounders related to demographic and clinical differences in patients of different cohorts, the main reason behind these contradictory findings could be attributed to the dose, timing, and form of VitD3 administered (i.e Calcifediol, cholecalciferol or calcitriol). For instance, Murai *et al*. reported that a single oral dose of 200,000 IU of vitamin D3 did not have a significant effect on the length of hospital stay [73]. Annweiler *et al*. demonstrated in their randomized controlled trial that the early administration of high-dose (400,000 IU cholecalciferol) versus standard-dose (50,000 IU) within 72 hours after the diagnosis of COVID-19 to at-risk older patients with COVID-19 improved overall mortality at day 14, but the effect was no longer observed after 28 days [75]. In our cohort, patients were given 50,000 IU cholecalciferol weekly for 2–3 weeks. Hence, factors such as timing, dosage, and form of VitD3 should be further investigated in a larger controlled cohorts to optimize the efficacy of VitD3.

In conclusion, the severity of SARS-CoV-2 infection was confirmed to be associated with NLRP3 inflammasome activation in our cohort. In turn, VitD3 supplementation suppressed inflammation in blood of severe COVID-19 patients, and this was associated with a reduction in disease severity and upregulation in type I IFN signaling. It is worth highlighting that our study focused on VitD3 as a supplement to ameliorate SARS-CoV-2 infection and did not treat selected patients based on their VitD3 deficiency status especially that the baseline serum levels of 25(OH)D concentrations of patients were not available and assigning patients to be administered or not with VitD3 was a mere physician decision following national guidelines of COVID-19 treatment. The correlation between VitD3 deficiency and COVID-19 severity will better stratify patients that are more likely to benefit from VitD3 supplementation. Despite these limitations, our study highlights that the clinically observed effect of VitD3 treatment during COVID-19 could be due, at least in part, to its suppression of NLRP3 inflammasome pathway; hence supporting its potential use as a supplement to ameliorate COVID-19 severity.

## Supporting information

S1 Table Gene expression datasets used in the study. (PDF)

S2 Table Primers sequence. (PDF)

S1 File Details of gene expression datasets used in the study. (XLSX)

S2 File S1-S4 Figs. Western blot images.
(ZIP)
