# Supplementary material for: Vitamin D regulates COVID-19 associated severity by suppressing the NLRP3 inflammasome pathway
Source: PLoS One. 2024 May 15;19(5):e0302818. doi: 10.1371/journal.pone.0302818 (PMC11095707; doi:10.1371/journal.pone.0302818)
Supplement: S1 Table — (PDF) [file pone.0302818.s001.pdf]

S1 Table. Gene expression datasets used in this study.

Groups	GEO accession	Platform	Sample	Condition 1	Condition 2
**RNA-seq Data**					
	GSE152075 (PMID: 32898168) [31]	GPL18573	Nasopharyngeal swabs (Human)	Negative controls (n=54)	COVID -19 (n=430)
	EGAS00001004503 (PMID: 33441124) [1]	GPL24676	Whole blood (Human)	Controls (n=10)	COVID -19 (n=39)
	GSE157103 (PMID: 33096026) [33]	GPL24676	Leukocytes from whole blood (Human)	Controls (non-COVID-19 patients) (n=10)	Non-severe COVID-19 (n=51), severe COVID-19 (n=37)
	GSE182264 (PMID: 35401811) [38]	GPL11002	Peritoneal macrophages (Mouse)	Peritoneal macrophages control (n=5)	Peritoneal macrophages transfected with Spike protein. (n=5)

	GSE208320 (PMID: 36341352) [36]	GPL28038	THP-1-derived macrophages (Human)	THP-1-derived macrophages controls. (n=2)	THP-1-derived macrophages transfected with Spike protein. (n=2)
	GSE169241 (PMID: 33853355) [37]	GPL24676	Human Embryonic stem cell (ES)derived macrophage	Mock infected human ES derived macrophages (n=3)	SARS-COV-2 infected human ES derived macrophages (n=3)
**Single-cell RNA-seq Data**					
	GSE145926 (PMID: 32398875) [34]	GPL23227	Bronchoalveolar lavage fluid (Human)	Healthy (n=6)	Moderate (n=3) and Severe (n=6) COVID-19
	GSE149689 (PMID: 32651212) [35]	GPL24676	Peripheral blood mononuclear cells (Human)	Healthy (n=4)	Severe Influenza (n=5), Severe COVID-19 (n=4), and Mild COVID-19 (n=4)
