# Supplementary material for: Vitamin D regulates COVID-19 associated severity by suppressing the NLRP3 inflammasome pathway
Source: PLoS One. 2024 May 15;19(5):e0302818. doi: 10.1371/journal.pone.0302818 (PMC11095707; doi:10.1371/journal.pone.0302818)
Supplement: S2 Table — (PDF) [file pone.0302818.s002.pdf]

S2 Table. Primers Sequence

	**Sequence (5'->3') Forward Primer**	**Sequence (5'->3') Reverse Primer**
NLRP3	CATGGCCGTGTGGATCTTCGCT	GTCTTCCTGGCATATCACAGTGGG
CASP-1	GGAGCTGAGGTTGACATCACAGGC	CAGAGGTCTTGTGCTCTGGGCG
IL-1β	ATGCACCTGTACGATCACTGA	ACAAAGGACATGGAGAACACC
IL-18	ATTGACCAAGGAAATCGGC	CTTCACAGAGATAGTTACAGC
18S	TGACTCAACACGGGAAACC	TCGCTCCACCAACTAAGAAC
