# Supplementary figures and images for: Vitamin D regulates COVID-19 associated severity by suppressing the NLRP3 inflammasome pathway
Source: PLoS One. 2024 May 15;19(5):e0302818. doi: 10.1371/journal.pone.0302818 (PMC11095707; doi:10.1371/journal.pone.0302818)

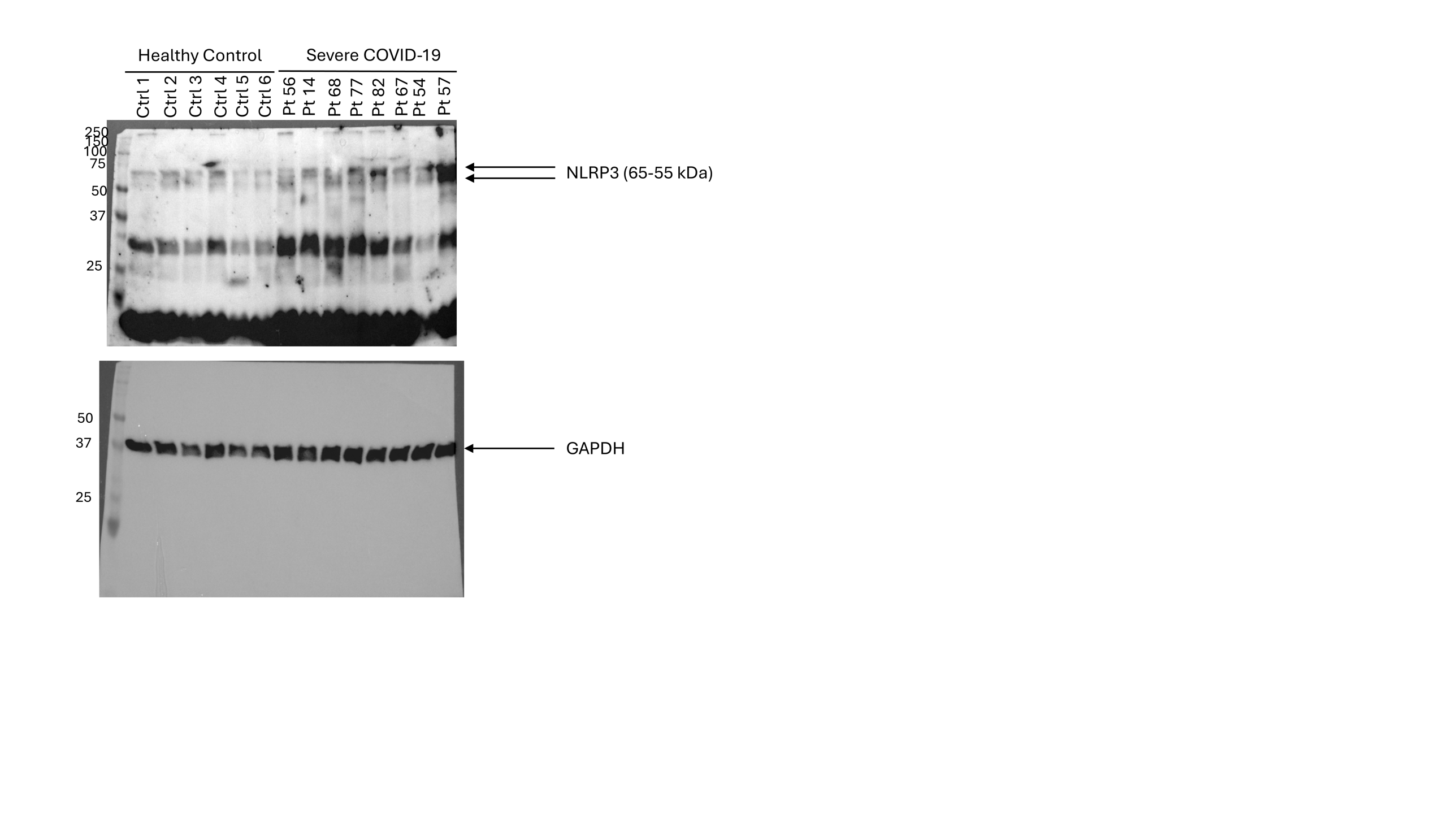

Supplement: S2 File — Western blot images. (ZIP) [file pone.0302818.s004.zip › Blot Images/S1 Fig, Tif.TIF]

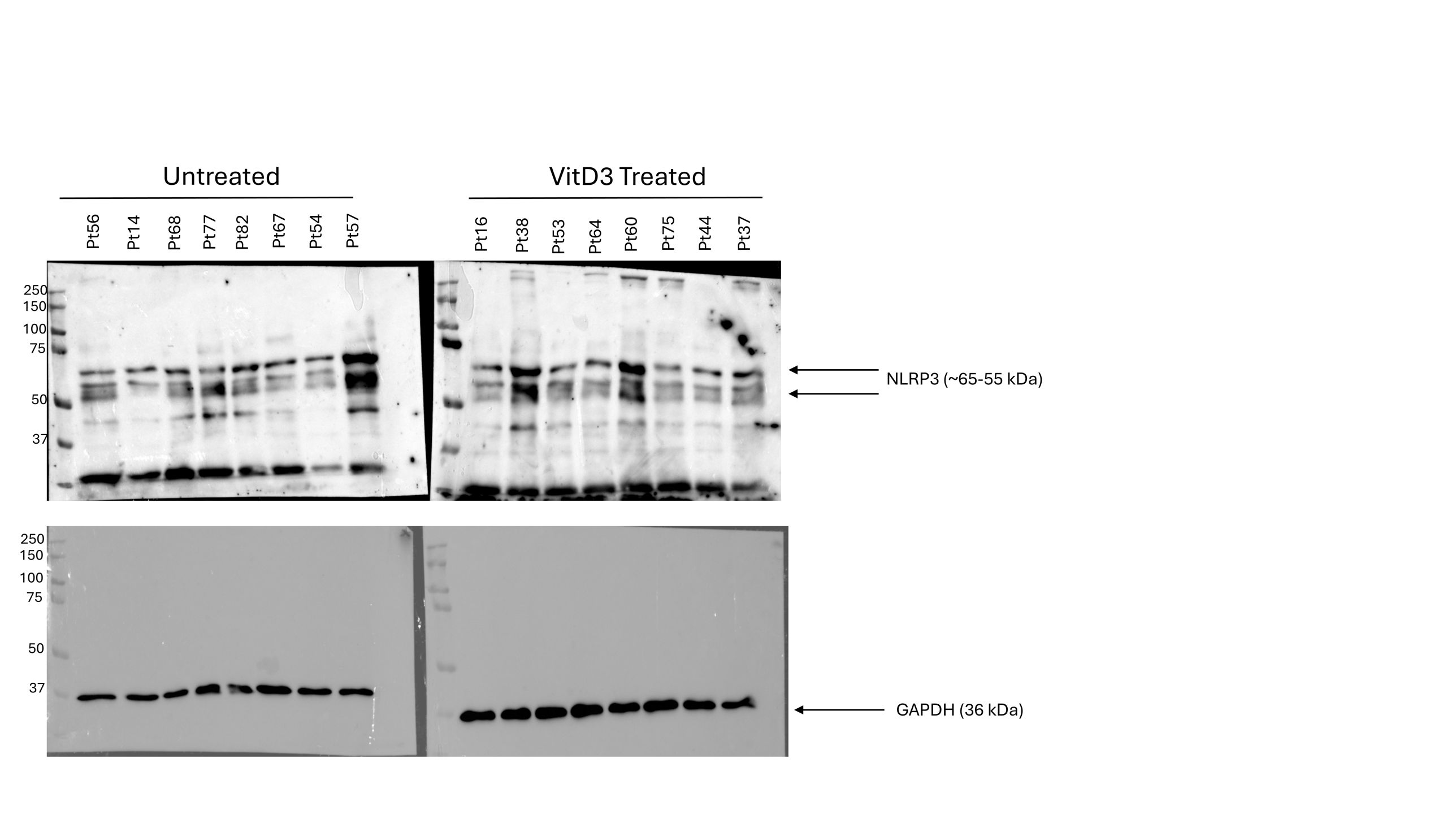

Supplement: S2 File — Western blot images. (ZIP) [file pone.0302818.s004.zip › Blot Images/S2 Fig. Tif.TIF]

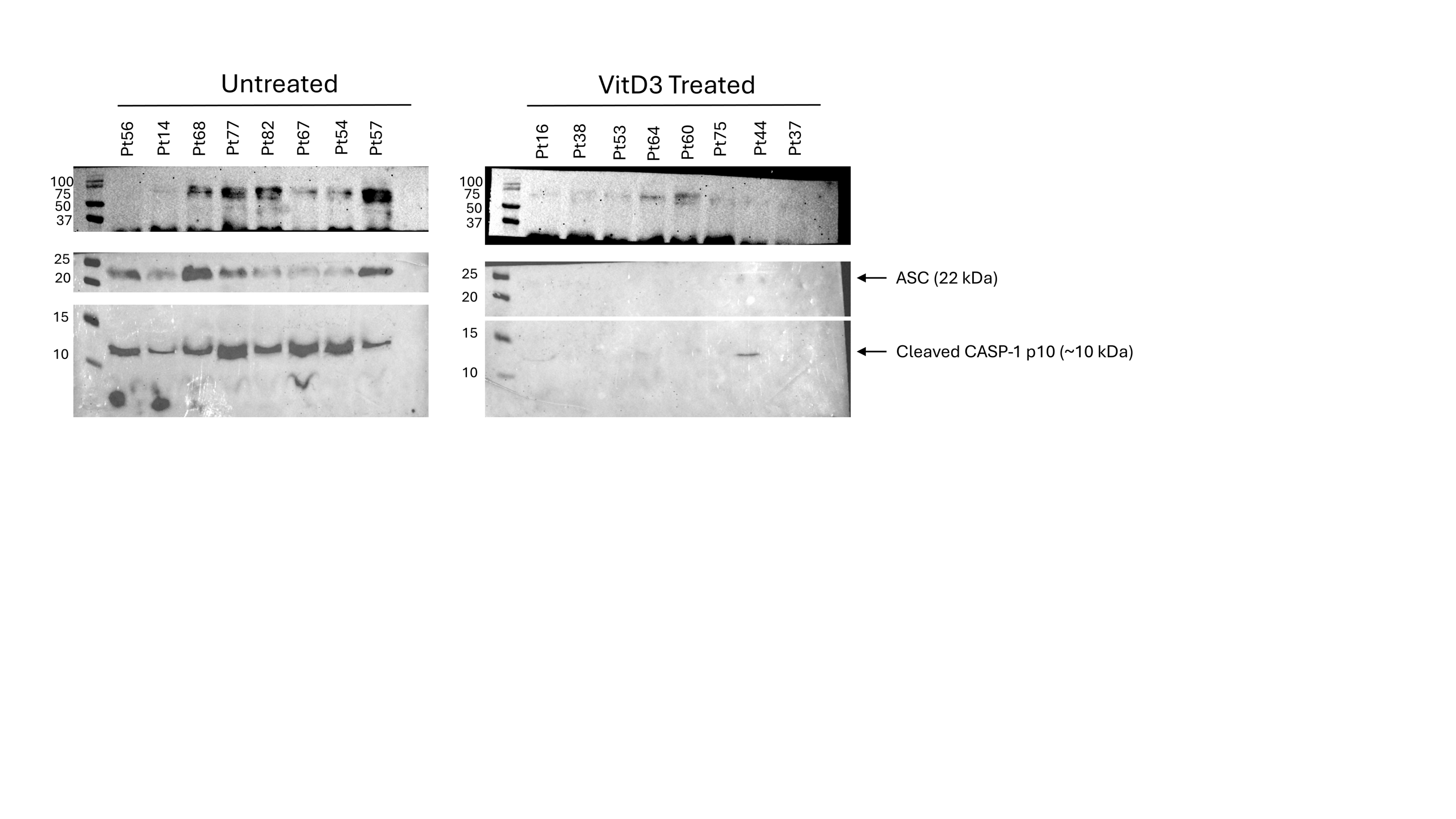

Supplement: S2 File — Western blot images. (ZIP) [file pone.0302818.s004.zip › Blot Images/S3 Fig. Tif.TIF]

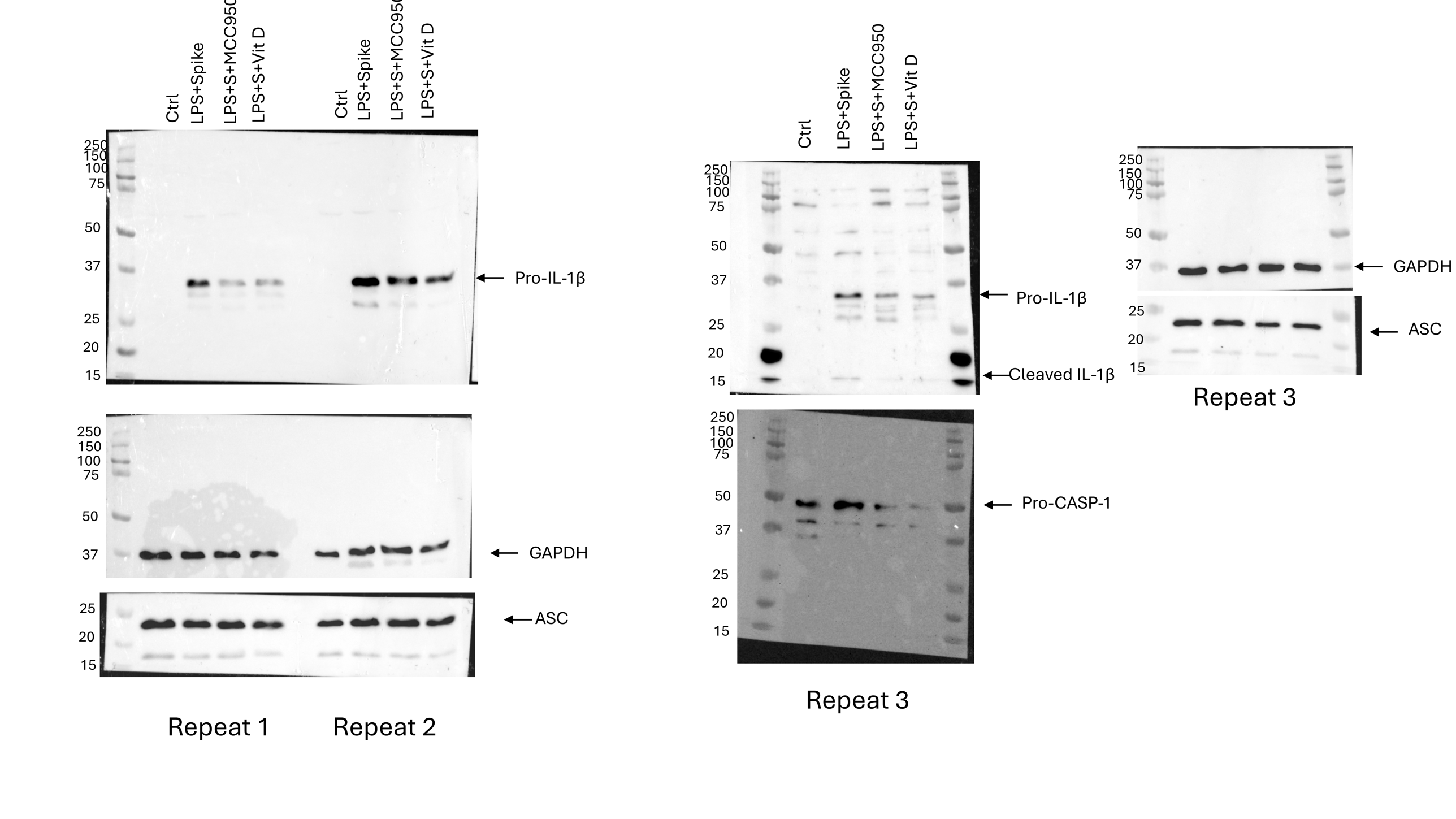

Supplement: S2 File — Western blot images. (ZIP) [file pone.0302818.s004.zip › Blot Images/S4 Fig. Tif.TIF]
